# Pulmonary Erdheim-Chester Disease With BRAF-AGAP3 Fusion: Late-Onset Osteolytic Femoral Lesions Despite Long-Term Pulmonary Stabilization With Corticosteroid

**DOI:** 10.7759/cureus.55670

**Published:** 2024-03-06

**Authors:** Koichi Nishino, Tatsuya Takagi, Takuo Hayashi, Shinya Kunimine, Hitoshi Tsuchihashi, Shunsuke Kato, Kazuhisa Takahashi, Kuniaki Seyama

**Affiliations:** 1 Department of Respiratory Medicine, Juntendo University Faculty of Medicine and Graduate School of Medicine, Tokyo, JPN; 2 Department of Orthopedics, Juntendo University Faculty of Medicine and Graduate School of Medicine, Tokyo, JPN; 3 Department of Human Pathology, Juntendo University Faculty of Medicine and Graduate School of Medicine, Tokyo, JPN; 4 Department of Dermatology, Juntendo University Faculty of Medicine and Graduate School of Medicine, Tokyo, JPN; 5 Department of Clinical Oncology, Juntendo University Faculty of Medicine and Graduate School of Medicine, Tokyo, JPN

**Keywords:** late-onset bone lesions, inhaled corticosteroids, trametinib, braf-agap3 fusion, erdheim-chester disease

## Abstract

Erdheim-Chester disease (ECD) is a rare inflammatory myeloid neoplasm affecting multiple systems and organs. The patient is a 38-year-old male with ECD complicated with pulmonary and cutaneous manifestations but without bone lesions diagnosed in 2008. Initial treatment with oral and inhaled corticosteroids achieved persistent favorable disease remission. However, atypical late-onset bone lesions developed in the bilateral femur in 2021. Although *BRAF-V600E* mutation was negative in the lung specimen at diagnosis, the next-generation gene sequence using biopsied bone lesions revealed a rare *BRAF-AGAP3 *fusion, leading to the administration of trametinib. This is the first report describing ECD harboring *BRAF-AGAP3 *fusion successfully treated with trametinib. Our case presents a unique clinical course in which late-onset osteolytic bone lesions developed despite a long-term stabilization of pulmonary lesions with low-dose oral and inhaled corticosteroids.

## Introduction

Erdheim-Chester disease (ECD) is a rare inflammatory myeloid neoplasm characterized by the infiltration of tissues by foamy CD68^+^CD1a^-^ histiocytes, which can affect multi-systems and organs such as bones, central nervous system, retroperitoneal organs, skin, lungs, cardiovascular system and endocrine [1-3]. Mutations activating the mitogen-activated protein kinase (MAPK) pathway are found in more than 80% of patients with ECD, mainly the *BRAF-V600E* mutation [4-6]. Many patients are diagnosed between the fifth and seventh decades of life, with male predominance [1-3]. Bone lesions are found in almost all patients with ECD typically presenting osteosclerosis [3]. Pulmonary lesions are commonly complicated with ECD, which can be progressive and result in poor prognosis of ECD [7]. Since the high prevalence of gene mutations altering the MAPK pathway in patients with ECD, the role of genetic evaluation, including a next-generation sequence (NGS), has become increasingly important which could lead to molecular-targeted therapies such as BRAF and MEK inhibitors [8-11].

In 2011, we reported a 38-year-old male patient with ECD characterized by progressive pulmonary and cutaneous manifestations but without bone lesions [12]. Although the role of systemic corticosteroids is generally limited to reducing edema or acute symptoms of ECD [13], we observed significant improvement and persistent remission by oral prednisolone after the last report. Additionally, we also experienced notable efficacy of inhaled corticosteroids on stabilizing pulmonary lesions. However, more than a decade after the diagnosis, the patient developed late-onset bone lesions which is an atypical presentation of ECD. A genetic re-evaluation of the bone lesions by NGS revealed a rare *BRAF-AGAP3* fusion, resulting in an effective trametinib treatment.

We herein present a case report of this unique case of ECD harboring *BRAF-AGAP3* fusion, exhibiting late-onset bone manifestations after prolonged stabilization of pulmonary lesions by oral and inhaled corticosteroids.

## Case presentation

As previously reported, the patient was diagnosed with ECD at the age of 38 in 2008 [12]. At the time of diagnosis, he presented both cutaneous (xanthelasma-like lesions on the eyelids and eruptions of the left temple) and pulmonary lesions (thickening of the pleura, interlobular septa, and peribronchovascular interstitial markings, as well as fine nodules and small cysts scattered predominantly in the bilateral lower lobes) without other organ involvements. Histopathological confirmation of ECD was made by video-assisted thoracoscopic surgery (VATS) of the lung, which revealed fibrous thickenings of the pleura and the interlobular septa along with marked infiltration of CD68^+^/Factor XIIIa^+^/S100^+^/CD1a^−^ foamy histiocytes in the fibrotic areas. Furthermore, multiple cysts surrounded by fibrous thickening of the pleura and interlobular septa were apparent, in which both alveoli and elastic laminas completely disappeared. *BRAF-V600E* mutation was not identified by polymerase chain reaction (PCR) of the obtained lung and skin tissues.

The initial treatment was started with an oral prednisolone dose of 30 mg/day, which was subsequently tapered to 3 mg/day over three years, resulting in a marked improvement in both pulmonary and cutaneous lesions. To successfully reduce the dose of systemic corticosteroids without exacerbation, inhaled budesonide (640 µg/day) and formoterol (18 µg/day) were added during prednisolone tapering. In addition to formoterol, inhaled tiotropium bromide (5 mg/day) was also initiated for exertional dyspnea, which was found to be effective.

**Figure 1:**
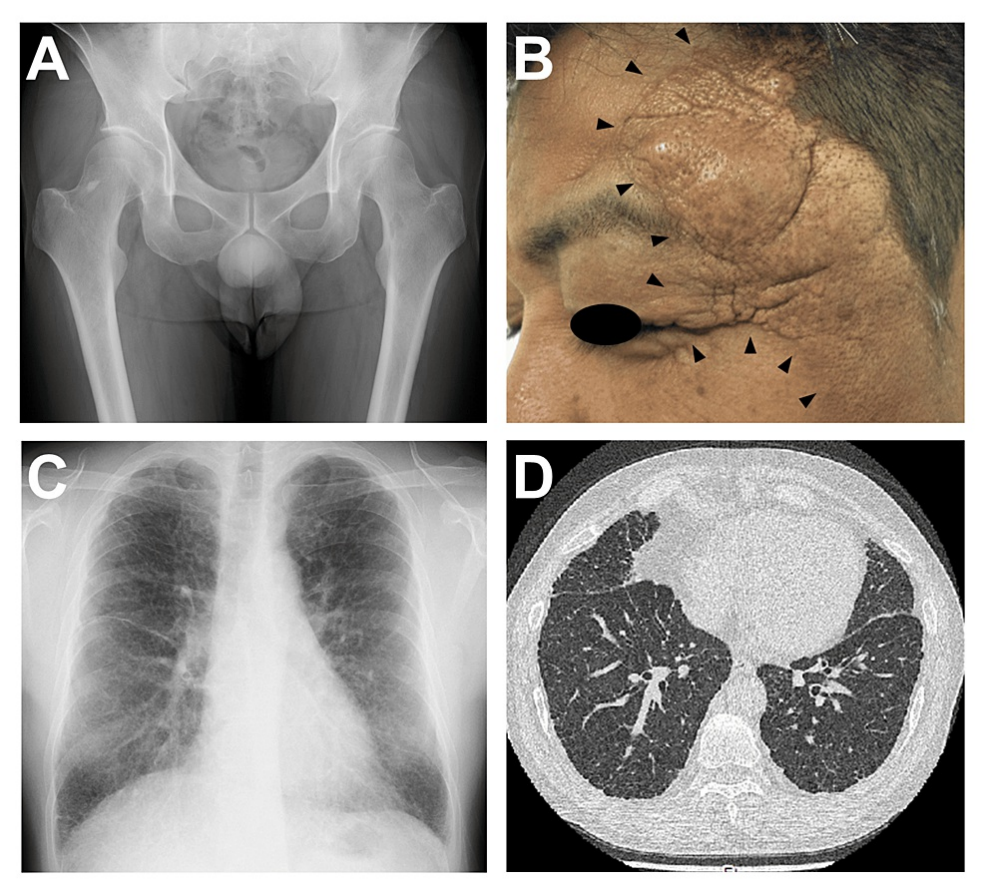
Clinical course following initial treatment with oral prednisolone, inhaled corticosteroids, and bronchodilators A. A hip joint radiograph taken after four years of treatment (2012) indicated no evidence of bone lesions. B. Despite an initial therapeutic response to prednisolone, cutaneous lesions on the left temple and eyelid (arrowheads) gradually exacerbated over the seven years of treatment (2015). C and D. Chest imaging taken seven years after the initial treatment (2015) showed significant improvement. A chest radiograph demonstrated thinning of minor fissures and a reduction of bilateral reticulonodular shadows (C). CT images of the chest displayed a reduction of the thickness of bilateral pleura and interlobular septa, along with a decrease in fine nodules and linear shadows. No apparent changes were observed in the scattered small cysts (D).

In 2012, the patient experienced mild pain around the left hip joint. A radiograph of the hip joint was unremarkable (Figure 1A), but hip joint effusion was noted by a local physician. Arthrocentesis was performed and the pain resolved spontaneously. In 2015, the cutaneous lesions on the left temple and eyelids gradually exacerbated, and monthly cryotherapy was initiated (Figure 1B). In contrast, pulmonary lesions were stabilized with a maintenance dose of prednisolone and inhalers (Figures 1C, 1D). Subsequently, the patient continued to receive these treatments on an outpatient basis.

**Figure 2:**
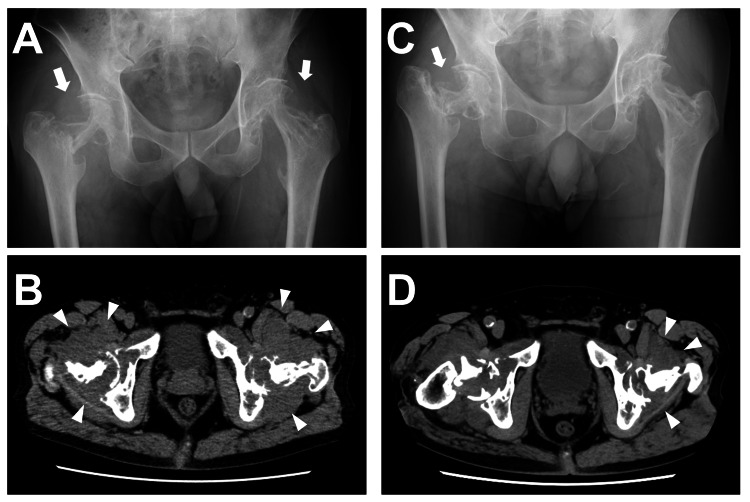
Changes in the hip joint lesions pre- and post-trametinib treatment The patient developed late-onset bone lesions in the femur 13 years following diagnosis (2021). A. A radiograph of the hip joint before trametinib therapy revealed osteolysis in the bilateral femoral necks (arrows). B. CT of the hip joint before trametinib administration showed bone destruction in both the femoral neck and the acetabulum. Additionally, soft-tissue density shadows were noted in the bilateral periarticular area (arrowheads). C. A radiograph of the hip joint one year after the trametinib treatment displayed shortening of the right femoral neck indicating fracture was evident (arrow). D. CT of the hip joint one year after starting trametinib showed a reduction of the periarticular lesions particularly on the left side (arrowheads).

**Figure 3:**
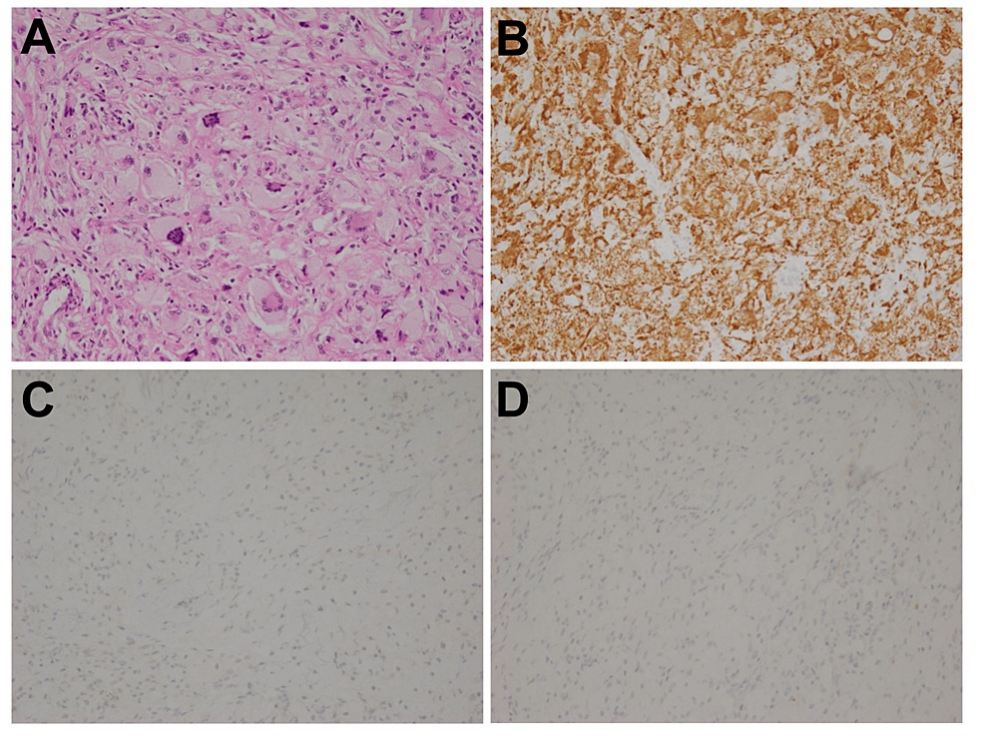
Histopathological findings of the biopsied specimen from the right hip joint A. Hematoxylin-eosin (HE) staining revealed a diffuse proliferation of foamy histiocytes and Touton giant cells, with infiltration of lymphocytes and proliferation of collagenous fibers (original magnification x 200). B-D. Immunohistochemistry demonstrated that foamy histiocytes were strongly positive for CD68 (B), but negative for CD1a (C) and S100 (D) (original magnification x 200).

In 2021, the patient paid a routine visit to the outpatient department and complained of worsening bilateral hip pain while walking. Physical examination revealed brown eruptions and xanthelasma-like lesions on the face. Chest auscultation was unremarkable. The range of motion in the hip joint was limited especially in extension and internal rotation. Tenderness was noted in the right Scarpa's triangle. A radiograph of the hip joint revealed osteolysis in the bilateral femoral necks (Figure 2A). CT of the hip joint showed bone destruction of the bilateral femoral neck and acetabulum, along with soft-tissue density shadows in the bilateral periarticular area (Figure 2B). A surgical biopsy was performed on the soft-tissue lesion in the right hip joint which was pathologically consistent with ECD (Figure 3). Notably, NGS revealed the ECD lesion of the hip harboring *BRAF-AGAP3* fusion.

In 2022, the patient was administered trametinib, a MEK inhibitor, at an initial dosage of 2 mg/day. Due to adverse events, including skin rash, diarrhea, liver dysfunction, and interstitial pneumonia, trametinib was temporally discontinued until the adverse events improved. Trametinib was resumed at a dose of 1 mg/day every other day. We confirmed that the patient could tolerate this dose of trametinib for six months, after which the dose was increased to 1 mg/day on a schedule of two days followed by one day off. This treatment regimen effectively managed pain in both hip joints, and the patient was able to maintain independence in his activities of daily living with crutches. An intramedullary nail was not considered to yield bone fixation, and prosthetic joint replacement involving the excision of periosteal tumors was deemed to carry a considerable risk of infection and dislocation.

**Figure 4:**
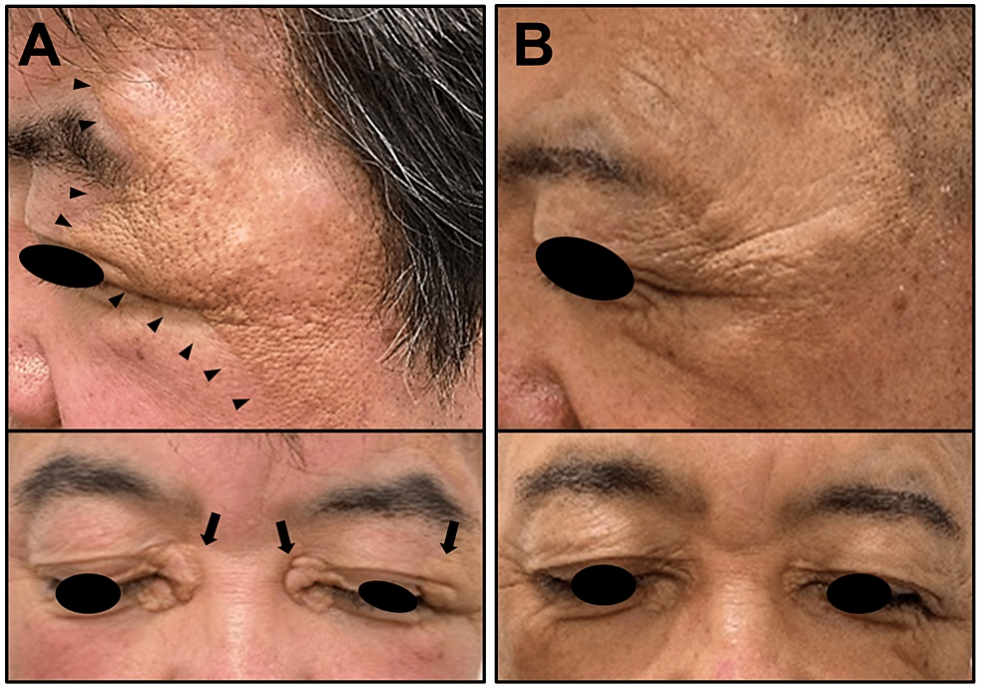
Improvement of cutaneous lesions after the initiation of trametinib treatment A. Cutaneous manifestations prior to trametinib treatment. Brown maculae accompanied by firm and elastotic eruptions were observed on the left temple (arrowheads). Xanthelasma-like lesions were also evident on both eyelids (arrows). B. Cutaneous manifestations one year after the initiation of trametinib treatment. A significant improvement was observed in the lesions on both the left temple and eyelids.

After one year of treatment with trametinib, a radiograph of the hip joint showed remaining osteolysis and fracture of the right femoral neck (Figure 2C). However, CT of the hip joint revealed the reduction of periarticular soft-tissue lesions, particularly on the left side (Figure 2D). There was also a notable improvement in brown eruptions on the left temple and xanthelasma-like lesions on the eyelids (Figure 4).

**Figure 5:**
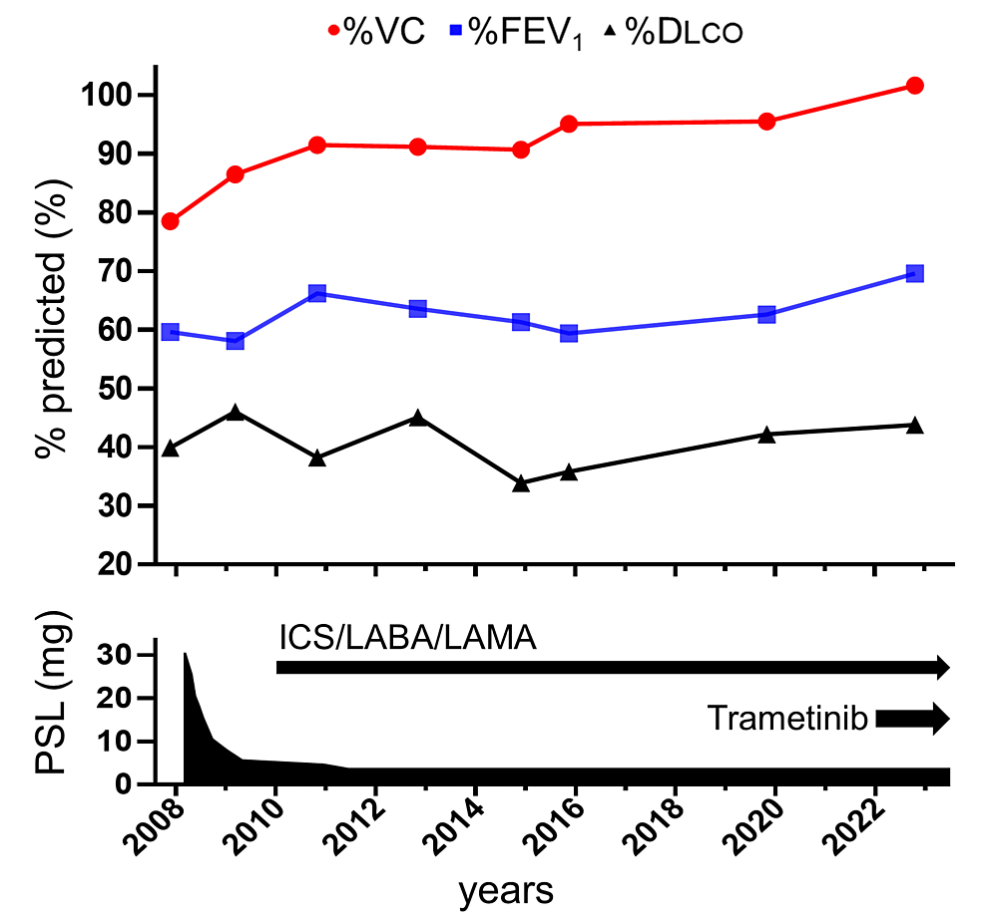
The course of pulmonary function and treatment Pulmonary function test at diagnosis showed moderate obstructive respiratory impairment along with a decreased diffusing capacity. Pulmonary function slightly improved following oral and inhaled corticosteroid therapy and was maintained throughout the course of treatment. Notably, pulmonary function further improved following the initiation of trametinib treatment. %DLCO: Diffusing capacity of carbon monoxide % of the predicted value; %FEV_1_: Forced expiratory volume in one second % of the predicted value; ICS: Inhaled corticosteroids; LABA: Long-acting β_2_-agonist; LAMA: Long-acting muscarinic antagonist; PSL: Prednisolone; %VC: Vital capacity % of the predicted value

Furthermore, amelioration of the pulmonary lesions was observed. A chest radiograph displayed increased translucency, while a CT of the chest revealed a reduction in reticular shadows and pleural septal thickenings. Although pulmonary function was maintained with low-dose prednisolone and inhalers throughout the clinical course, an additional improvement in pulmonary status was observed following the induction of trametinib (Figure 5). C-reactive protein was decreased from 11.06 mg/dL to 0.56 mg/dL by trametinib treatment. Overall, the patient demonstrated a partial response to trametinib and has been able to continue trametinib for one and a half years without any major adverse events.

## Discussion

Bone lesions are the most common manifestation of ECD, observed in up to 96% of patients [3]. The most frequently affected bones are the long bones such as the femur and tibia rather than the skull or vertebrae [14]. Osteosclerosis is a typical presentation of bone lesions, however, osteolysis is only observed in 5-30% of patients, as described in our case [14]. The mechanism underlying the development of late-onset bone lesions is currently unknown. According to Estrada-Veras et al., most ECD patients without bone lesions in their study had already received some sort of therapy at evaluation [2]. Therefore, early therapeutic intervention may delay the development of bone lesions. In our case, the early diagnosis at a young age and the initial treatment likely played a crucial role in delaying the development of bone lesions. Unfortunately, advanced late-onset bone lesions arose in our patient more than 10 years after his initial diagnosis. The current consensus recommendation suggests performing regular imaging examinations including 18 F-fluorodeoxyglucose positron emission tomography-computed tomography (FDG-PET-CT) scan, whole-body CT, or 99mTc bone scintigraphy for ECD [13]. Overall, the present case illustrates the importance of these periodic imaging studies for the detection of late-onset bone lesions even in patients with ECD with no bone lesions at diagnosis and who showed prolonged stabilization of the initial lesions by treatment thereafter.

Pulmonary manifestations commonly occur in ECD, affecting 18% to 52% of patients, and are often asymptomatic [1-3,15]. CT of the chest may present interlobular septal thickening, pulmonary nodules, airway thickening, ground glass opacities, pleural thickening, and mediastinal infiltration [15-17]. In most cases, pulmonary function is normal; however, a restrictive pattern is typically more common than an obstructive one, often accompanied by reduced diffusing capacity for carbon monoxide [2,15]. In contrast to the typical presentation of pulmonary ECD, our patient showed a significant obstructive pattern on spirometry. The exact causes of obstructive change in pulmonary ECD remain unclear, however, one possible reason is that the ECD-related pulmonary lesions themselves may be responsible for obstructing the peripheral airways. Indeed, in our case, CT of the chest showed thickening of bronchovascular bundles and biopsied lung specimens revealed multiple cystic distractions and loss of alveoli within the ECD lesion [12]. These changes in the lungs could lead to the narrowing or collapse of peripheral airways, resulting in airflow limitation. Another possible mechanism could involve coexisting chronic obstructive pulmonary disease (COPD), potentially related to the patient's smoking history (30 pack-years). However, considering the patient's relatively young age of 38 at diagnosis and the lack of evident emphysematous changes in both the chest CT and VATS specimen, it seems unlikely that COPD is the main cause of the observed moderate obstructive change.

Gene mutations activating the MAPK pathway are found in most patients with ECD. Since* BRAF-V600E* mutations are observed in approximately 50% of patients, testing for *BRAF-V600E* should be pursued for all patients with ECD [13]. In cases without *BRAF-V600E* mutation, NGS should be considered to detect other mutations in MAPK or mechanistic targets of the rapamycin (mTOR) pathway [13]. Indeed, our patient’s lung and skin specimen at diagnosis was negative for *BRAF-V600E *mutation by PCR. However, NGS analysis using the late-onset bone lesion revealed* BRAF-AGAP3 *fusion, leading to the effective targeted therapy with trametinib. The prevalence of BRAF fusion in ECD was very rare; only 4% (2 out of 46 patients) were found to harbor *BRAF-UBTD2* and *BRAF-RNF11 *fusion [18]. Regarding ECD with *BRAF-AGAP3 *fusion, a review of the literature revealed no prior reports.

Before the emergence of targeted therapy, IFN-α and PEG-IFN-α were considered conventional treatments for ECD that were able to achieve significant improvement in survival [13,19]. To date, BRAF inhibitors such as vemurafenib or dabrafenib are recommended as the first-line treatment for ECD patients with *BRAF-V600E* mutations [13]. For patients without* BRAF-V600* mutations, MEK inhibitors including cobimetinib or trametinib have been shown to be effective [9,11]. Trametinib demonstrated a 59% partial response rate and a 12% complete response rate for patients with ECD [20]. In this study, most patients required lower than standard doses of trametinib due to adverse events including skin rash and diarrhea, but were responsive to lower doses (0.5-1 mg/day), which was similar to our case [20].

Currently, systemic corticosteroids may be used to relieve acute symptoms or edema but are not considered an effective monotherapy for ECD [13]. Regarding inhaled corticosteroids, there has been no clinical evidence to suggest their effect on pulmonary ECD. However, our patient had durable benefits from corticosteroids; improvement and stabilization of pulmonary lesions were achieved for more than 10 years. In particular, the marked discrepancy in the clinical behaviors between the pulmonary and cutaneous manifestations strongly suggests that inhaled corticosteroids exerted certain beneficial effects on pulmonary lesions in our patient. Although the exact mechanism is unknown, *BRAF-AGAP3* fusion may contribute to the notable sensitivity of the abnormal histiocytes to corticosteroids, which may not be seen in *BRAF-V600E* mutations. We further speculate that the hydrophilic property of budesonide might allow it to distribute to various sites where abnormal histiocytes proliferate in the lungs. In addition, corticosteroid administration at a very early stage of ECD may have been effective in preventing irreversible lung damage in our patient. Given the additional improvement in pulmonary function by trametinib achieved in our patient, we suggest that corticosteroids may act as a disease stabilizer rather than a curative treatment. Further studies are warranted to evaluate the characteristics of cases of ECD harboring BRAF-fusion, including the advantages of oral and inhaled corticosteroids.

## Conclusions

Herein, we reported a case of ECD harboring a rare* BRAF-AGAP3* fusion that responded well to trametinib. This report is peculiar because we observed a very long clinical course of ECD, while the patient exhibited unusual late-onset bone manifestations following an extended period of stabilization of pulmonary lesions. Our case illustrates the importance of persistent genetic sequences to detect MAPK or mTOR pathway mutations in ECD, even though the initial gene analysis was negative for *BRAF-V600E*. In addition, a periodic survey of the whole body is critical in managing ECD to detect late-onset manifestations in various organs. Although the role of corticosteroids is generally limited to transient symptom relief, our patient showed a marked response to corticosteroids. The present report raises the possibility that oral and inhaled corticosteroids could offer therapeutic benefits for certain types of pulmonary ECD, including *BRAF-AGAP3* fusion. Early administration of corticosteroids may further help to stabilize the disease progression of ECD over a long period.
